# Synergistic Optimization of Microstructure and Mechanical Properties of 7075 Aluminum Alloy Sheet via Controlling Rolling Passes and Pass Reduction

**DOI:** 10.3390/ma19030479

**Published:** 2026-01-25

**Authors:** Xiaodong Zhang, Jufu Jiang, Jian Dong, Ying Wang, Jingbo Cui, Lingbo Kong

**Affiliations:** 1 School of Materials Science and Engineering, Harbin Institute of Technology, Harbin 150001, China; 25b309040@stu.hit.edu.cn (X.Z.); 22b909131@stu.hit.edu.cn (J.D.); cuicuijingbo@163.com (J.C.); 24b909018@stu.hit.edu.cn (L.K.); 2 National Key Laboratory for Precision Hot Forming, Harbin Institute of Technology, Harbin 150001, China; 3 School of Mechatronics Engineering, Harbin Institute of Technology, Harbin 150001, China

**Keywords:** 7075 alloy, hot-rolled, microstructure, mechanical properties, texture

## Abstract

The pass reduction in hot rolling significantly influences the properties of 7075 alloy sheets, yet its quantitative effect requires systematic investigation. Multi-pass hot rolling experiments with 11% and 16% pass reductions were conducted on forged 7075 alloy. The microstructure, texture evolution, and mechanical properties were analyzed using SEM, EBSD, and mechanical testing. As the total thickness reduction increased, a clear correlation was observed with the enhanced mechanical properties of the hot-rolled 7075 alloy, demonstrated by the concurrent rise in both ultimate tensile strength (UTS) and yield strength (YS). When the total reduction exceeded 60%, the strengthening effect was most pronounced, with UTS and YS reaching 367.09 MPa and 332.82 MPa, respectively. The average grain sizes of 31.49 μm and 27.56 μm were achieved at the 12th pass (11% reduction per pass) and the 8th pass (16% reduction per pass), respectively. Under the condition of 11% reduction per pass, the texture intensity exhibited a non-monotonic trend with increasing passes. T6, T7, and RRA heat treatments were applied to the final rolled plates, and the maximum mechanical properties obtained in the hot-rolled 7075 plate following T6 heat treatment were UTS of 607.5 MPa, YS of 580.9 MPa, and elongation of 13.6%.

## 1. Introduction

Nowadays, high-strength aluminum alloy sheets, owing to their excellent specific strength, are extensively utilized in components such as fuselage skin panels, bulkheads, and support pillars of automobiles [1,2]. Flat-rolled aluminum products, especially high-strength 7xxx series alloys such as 7075 aluminum alloy, have been widely used as key structural materials in aerospace, automotive, and battery fields due to their excellent specific strength, good toughness, and fatigue resistance [3,4,5,6]. The performance of these alloys is fundamentally determined by their thermomechanical processing history. Hot rolling serves as an essential fabrication step for achieving high-performance plates and sheets, where the pass reduction is a critical yet underexplored parameter. It plays a decisive role in governing dynamic recrystallization, texture evolution, and the resultant mechanical properties [7,8,9,10]. Recent studies have employed integrated numerical and experimental approaches to explore hot rolling processes. Zhang et al. [11] investigated snake hot rolling through finite element simulation and reported that speed ratio and offset significantly affected shear strain penetration toward the mid-thickness region, improving deformation uniformity. Other studies on accumulative roll bonding (ARB) indicated that interlayer interfacial stress promoted the formation of brass texture components, and the critical strain for necking in hard layers was closely related to the layer thickness ratio [12]. Wang et al. [13] proposed a multiscale simulation method, introducing a recrystallized grain model into hot rolling simulations and providing a novel approach for analyzing dynamic recrystallization behavior during complex thermo-mechanical processes. Accurate prediction of hot rolling forces was realized in the work of Shang et al. [14] via a novel framework that fused multi-channel deep learning, physical mechanisms, reinforcement learning tuning, and anomaly correction techniques. J.H. Driver et al. [15] established through combined experimental and simulation studies that the high strength of hot-rolled aluminum sheets under high-temperature and high-strain conditions arises from the formation of a pronounced brass texture. Zeng et al. [10] reported that the texture evolution of AA5052 aluminum alloy in the continuous casting state during single-pass hot rolling, and found that grain microstructure changed from equiaxed grains to elongated grains with a reduction of over 38%.

Hot rolling is a critical manufacturing process that not only enables precise dimensional control and high production efficiency [16,17] but, more importantly, directly determines the final microstructure and properties of the sheet [18,19]. Within this process, the pass reduction is a fundamental parameter. It governs the degree of plastic deformation, which enhances strength through work hardening and grain refinement. By increasing the pass reduction during hot rolling, more intense work hardening is induced in the sheet, thereby significantly enhancing its mechanical properties. However, if not properly managed, excessive unidirectional deformation can promote the formation of fibrous, elongated grains, leading to strong mechanical anisotropy [20,21]. By adjusting the number of rolling passes and the pass reduction to appropriate levels, this anisotropy can be mitigated to some extent. Meanwhile, dynamic recrystallization (DRX) is also a key mechanism to counteract this anisotropy, which is activated during hot rolling. DRX effectively refines the grain structure and can simultaneously improve both strength and ductility [21,22]. The extent and characteristics of DRX, and thus the final recrystallized microstructure, are highly sensitive to hot rolling parameters. Numerous studies have adopted the method of controlling rolling temperature and reduction ratio to regulate the microstructural evolution during recrystallization [23,24,25,26]. Despite this understanding, a significant research gap remains: there is a lack of systematic, quantitative studies on how pass reduction during multi-pass hot rolling specifically influences the microstructural gradient, texture evolution, and strength–ductility balance in highly alloyed plates like 7075 aluminum alloy [27,28,29,30,31]. To address this gap, the present work is designed to clarify the specific role of pass reduction. We conducted hot rolling experiments on forged 7075 alloy using two distinct pass reduction schedules (11% and 16%). By combining detailed EBSD texture analysis with comprehensive mechanical testing, this study aims to elucidate the influence of pass reduction and rolling passes on DRX behavior, grain orientation evolution, and the resultant strengthening-toughening mechanisms. The findings are expected to provide a theoretical basis for optimizing the rolling process of high-performance aluminum sheets.

## 2. Materials and Methods

### 2.1. Preparation of Raw Materials

**Figure 1:**
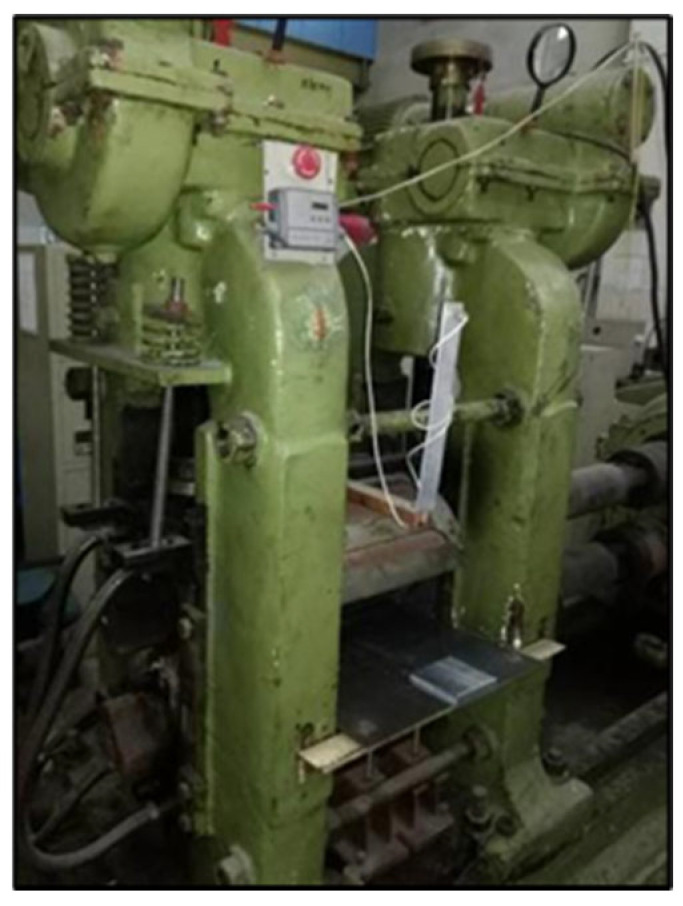
Laboratory two-roll rolling mill.

The experimental material was obtained from a commercial 7075 alloy in the as-forged state. Its chemical composition was determined by Inductively Coupled Plasma (ICP) analysis, and the results are presented in Table 1. This material was subsequently machined into a block measuring 105 × 98 × 21 mm^3^. As shown in Figure 1, the hot rolling trials were conducted using a laboratory rolling mill equipped with two rolls of 220 mm in diameter and 360 mm in length.

The as-forged 7075 aluminum alloy billet was heated to 465 °C and held for 8 h in a chamber resistance furnace to homogenize the internal microstructure, allowing the grains to revert from an elongated state to an equiaxed shape before the first rolling pass. After the initial pass, the 7075 billet was reheated to 400 °C, held for 1 h, and then subjected to subsequent rolling passes. To investigate the effects of different pass reductions and total reduction ratios on the microstructure and mechanical properties, two experimental schemes were designed for the 7075 billets with pass reductions of 11% (P1) and 16% (P2), respectively. The specific experimental parameters are shown in Table 2 and Table 3. The first rolling pass was all set at a 5% reduction in the schemes P1 and P2. The pass reduction is about 11% during the 2nd and 10th pass in the scheme P1. The pass reduction is about 16% during the 2nd and 15th pass in the scheme P2. Schemes P1 and P2 consisted of 15 and 10 passes, respectively, both achieving a final thickness of 4 mm. Following each pass, the plate was held at 400 °C for 5 min prior to subsequent rolling. Upon achieving the target reduction, it was air-cooled to room temperature, after which the final thickness was measured with a vernier caliper, and the average of 10 measurements was taken as the thickness value. The rolling mill speed was adjustable via a frequency conversion device. The temperature of the rolled sheet was measured using a contact temperature tester.

### 2.2. Microstructure and Performance Testing

**Figure 2:**
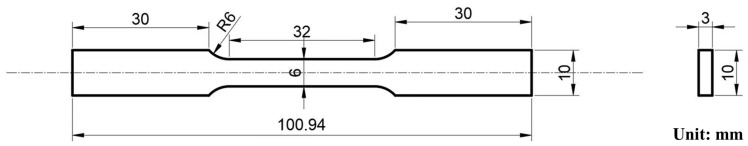
Schematic diagram of tensile specimen size.

Tensile tests were conducted on an AG-X Plus 250 kN universal material testing machine at a constant speed of 1 mm/min. Tensile specimens were fabricated along the rolling direction (RD) and the transverse direction (TD) from the hot-rolled 7075 alloy sheets according to ASTM-E8M Standard Test Methods [32]. The specific dimensions of the tensile specimens are shown in Figure 2. Five tensile specimens were tested under identical conditions to calculate standard deviation. Fracture surfaces of the tensile specimens were examined using a Quanta 200FEG field emission scanning electron microscope (SEM) (FEI, Harbin, Heilongjiang Province, China). Microstructure specimens were sectioned from the hot-rolled 7075 alloy, ground progressively with 200, 400, 800, 1200, 2000, and 3000 grit sandpapers, and then electropolished using a mixture of perchloric acid and ethanol (10 mL of 70 vol% HClO_4_ and 90 mL of C_2_H_6_O). Electropolishing was performed at 20 V and 0.3 A for 35 s. The microstructure of the hot-rolled 7075 alloy was initially examined using an optical microscope (OM). For a detailed assessment of crystallographic characteristics, Electron Backscatter Diffraction (EBSD) analysis was performed on samples that were ultrasonically cleaned for 5 min, using a Quanta 200FEG field emission scanning electron microscope equipped with an orientation analysis system.

## 3. Results and Discussion

### 3.1. Effects of Pass Reduction on Forged Hot-Rolled Plate Mechanical Properties

**Figure 3:**
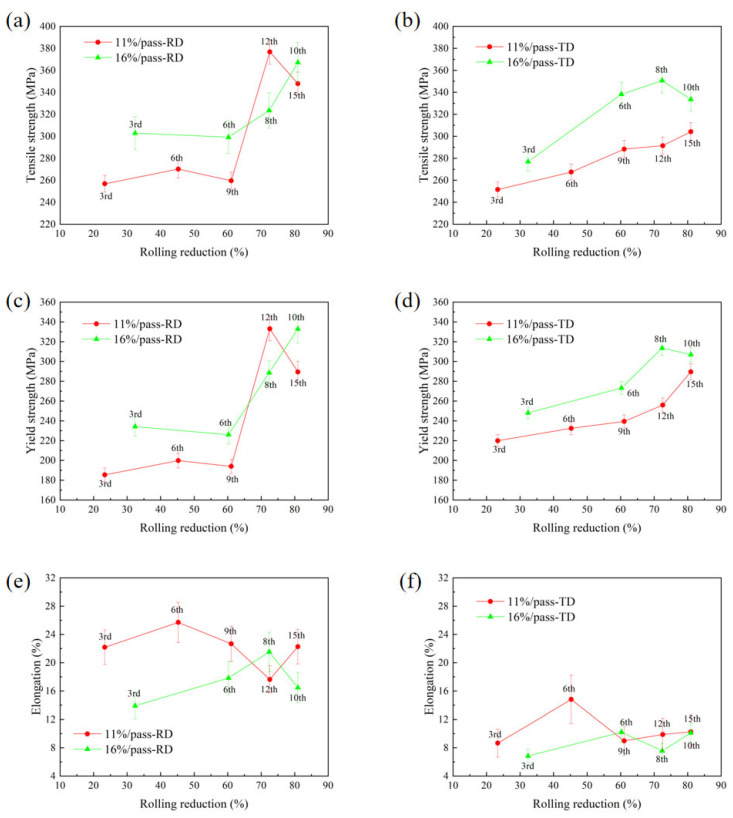
Variation in mechanical properties with roll reduction for hot-rolled forged 7075 alloy: (**a**) and (**b**) UTS; (**c**) and (**d**) YS; (**e**) and (**f**) Elongation. (Error bars represent standard deviation (*n* = 5)).

Figure 3 depicts the mechanical properties of the forged hot-rolled 7075 alloy tested along the rolling direction (RD) and transverse direction (TD) under two pass reductions (11% and 16%). A consistent strengthening trend with increasing rolling passes is observed for all conditions. Key variations in the mechanical properties are presented in Table 4.

The primary strengthening is attributed to work hardening from accumulated plastic deformation, which raises dislocation density and impedes slip. However, the extent of strengthening and the anisotropy between RD and TD are directly governed by the pass reduction and total number of passes. Specifically, the higher 16% pass reduction imposes more intense strain per pass. This not only enhances work hardening but also more effectively promotes dynamic recrystallization (DRX) and grain refinement at higher cumulative strains (e.g., by the 10th pass), contributing to the strength increase. Concurrently, this pronounced deformation accelerates the evolution of crystallographic texture. The stronger initial texture formation, followed by its randomization through DRX at high strains, is responsible for the significant improvement in TD ductility (from 6.3% to 10.5%) observed in the 16% schedule, thereby reducing the RD-TD anisotropy. In contrast, the 11% reduction schedule, with its milder strain per pass, likely results in a slower and less complete microstructural evolution. This analysis clarifies that the pass reduction is not merely a process parameter but a critical factor controlling the competition and synergy between work hardening, recrystallization, and texture evolution, ultimately dictating the final mechanical property profile.

Furthermore, a marked enhancement in mechanical properties was observed when the total rolling reduction exceeded 60%. Specifically, progressive rolling from the 9th pass to the 15th pass under an 11% per-pass reduction resulted in increases of 87 MPa in ultimate tensile strength and 91 MPa in yield strength, while rolling from the 6th pass to the 10th pass under a 16% per-pass reduction yielded increases of 74 MPa in ultimate tensile strength and 108 MPa in yield strength. Consequently, a significant strengthening effect was achieved in the rolling direction for both processing conditions. Wang et al. found that within the alloy material during deformation, the initial strain distribution was relatively homogeneous. Significant plastic deformation commenced only under conditions of substantial imposed strain. Large deformations exceeding 60% resulted in extremely high cumulative strain, a pronounced increase in dislocation tangles and pile-ups, and a sharp increase in the resistance to dislocation motion. Consequently, both the yield strength and tensile strength were substantially enhanced. During hot rolling, intense plastic deformation induces the development of preferred crystallographic orientations, leading to texture formation. Due to variations in the deformation processes and constraint conditions along different directions, distinct texture components evolve in different orientations. Consequently, this results in pronounced anisotropy in the mechanical properties across different rolling directions. While hot rolling of cast-state plates typically results in significant property differences between the Transverse Direction (TD) and Rolling Direction (RD) [33], the present study observed a less pronounced discrepancy in hot-rolled forged-state 7075 aluminum plates. Specifically, under a 16% per-pass reduction, the maximum tensile strength reached 371 MPa in the RD and 338 MPa in the TD. This reduced anisotropy can be attributed to the initial forged state of the material. The prior multi-directional forging deformation subjected the grains to complex strain paths, fostering the development of a more randomized texture, lacking the strong preferred orientations typically found in cold-rolled sheets [34]. Consequently, the anisotropy in mechanical properties between the TD and RDs was significantly diminished.

### 3.2. Effect of Pass Reduction on the Microstructure of Hot-Rolled Forged 7075 Alloy

**Figure 4:**
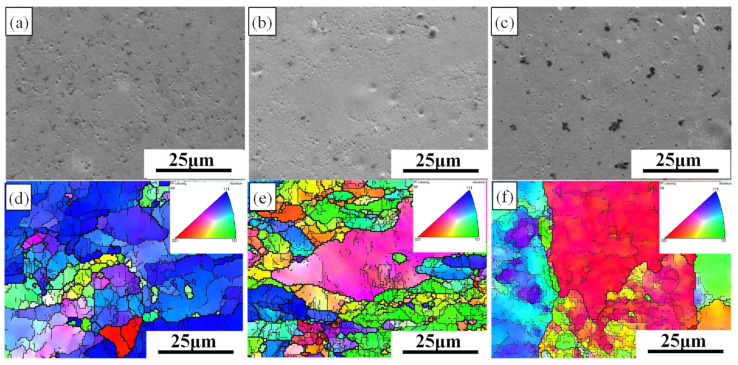
SEM and IPF images of 11% pass reduction rolling: (**a**) SEM image of 9th pass; (**d**) IPF image of 9th pass; (**b**) SEM image of 12th pass; (**e**) IPF image of 12th pass; (**c**) SEM image of 15th pass; (**f**) IPF image of 15th pass.

**Figure 5:**
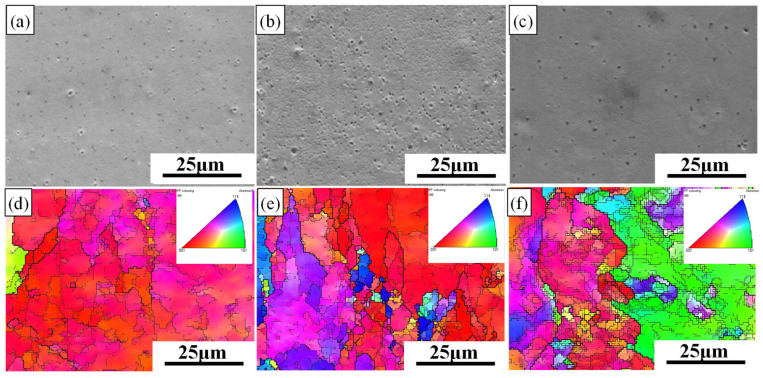
SEM, IPF images of 16% pass reduction rolling: (**a**) SEM image of 6th pass; (**d**) IPF image of 6th pass; (**b**) SEM image of 8th pass; (**e**) IPF image of 8th pass; (**c**) SEM image of 10th pass; (**f**) IPF image of 10th pass.

Figure 4 and Figure 5 show the scanning electron microscope (SEM) morphology and the layered microstructure inverse pole figure (IPF) maps of the rolled 7075 aluminum alloy sheets under different pass reductions, respectively. As the number of rolling passes increases, the initially coarse as-forged grains undergo progressive refinement, as evidenced in the early-pass microstructures (Figure 4a,d and Figure 5a,d). A distinct microstructural evolution is observed at intermediate strains, which corresponds to specific pass numbers under each schedule: after the 12th pass (11% reduction) and the 8th pass (16% reduction). At these stages, plastic deformation promotes a gradual transition from equiaxed to columnar grain shapes, accompanied by the development of a preferred crystallographic orientation (Figure 4b,e and Figure 5b,e). The accumulated strain further drives microstructural changes, notably causing intense dislocation pile-ups and strain gradients around second-phase particles. These sites, in turn, act as preferential nucleation points for subsequent recrystallization, effectively increasing the grain boundary area. Upon reaching maximum deformation (final pass), enhanced dynamic recrystallization (DRX) becomes dominant. This leads to the formation of new, finer equiaxed grains and results in slight grain growth in the most heavily strained regions (Figure 4c,f and Figure 5c,f). The final microstructure is therefore a mixture of deformed and recrystallized regions.

This evolution directly improves mechanical properties. Quantitative analysis of EBSD data indicates that the process reduces overall dislocation density and sub-grain content while promoting equiaxed grain formation. Consequently, the proportion of low-angle grain boundaries (LAGBs) decreases, while that of high-angle grain boundaries (HAGBs) increases [35,36], consistent with the progression of recovery and recrystallization.

**Figure 6:**
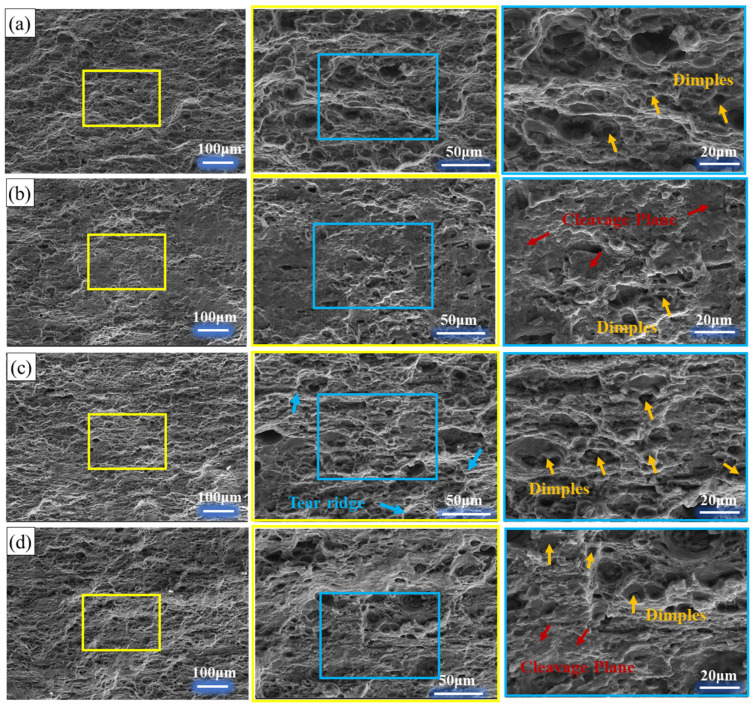
Tensile fracture morphology under different rolling directions and pass reductions: (**a**) RD-11%; (**b**) TD-11%; (**c**) RD-16%; (**d**) TD-16%.

The tensile fracture morphology of the hot-rolled 7075 alloy under 11% and 16% pass reductions is presented in Figure 6. All specimens exhibit surfaces dominated by abundant dimples, confirming that ductile fracture is the primary failure mode. This results from the nucleation, growth, and coalescence of microvoids, a process initiated by stress concentration at second-phase particles during deformation. A clear anisotropy in fracture morphology is observed between the RD and TD. As shown in Figure 6a,c for the RD, the dimples are more numerous and deeply elongated, aligned with the material flow. In contrast, the TD specimens (Figure 6b,d) show relatively shallower dimples alongside distinct cleavage platforms. This indicates a mixed-mode fracture in the TD, with reduced ductility.

This anisotropy is directly linked to the microstructural directionality induced by hot rolling. The microstructure, characterized by elongated grains and textured alignment (see Figure 4 and Figure 5), offers greater resistance to void growth along the RD. Conversely, cracking propagates more easily across the grain flow in the TD, facilitating cleavage. Furthermore, the degree of this anisotropy is influenced by the pass reduction. The specimen processed with the higher 16% reduction (Figure 6c,d) exhibits a more pronounced difference between RD and TD morphologies. This is consistent with the stronger texture and more elongated grain structure developed under the more severe 16% per-pass deformation, as previously discussed. The 11% reduction condition (Figure 6a,b) results in a comparatively less severe anisotropic fracture appearance. In summary, while ductile fracture is predominant, the fracture path and morphology are highly direction-sensitive. The anisotropy stems from the directional microstructure, and its severity is amplified by a higher pass reduction (16%), which promotes stronger texture and grain elongation compared to the 11% reduction schedule.

**Figure 7:**
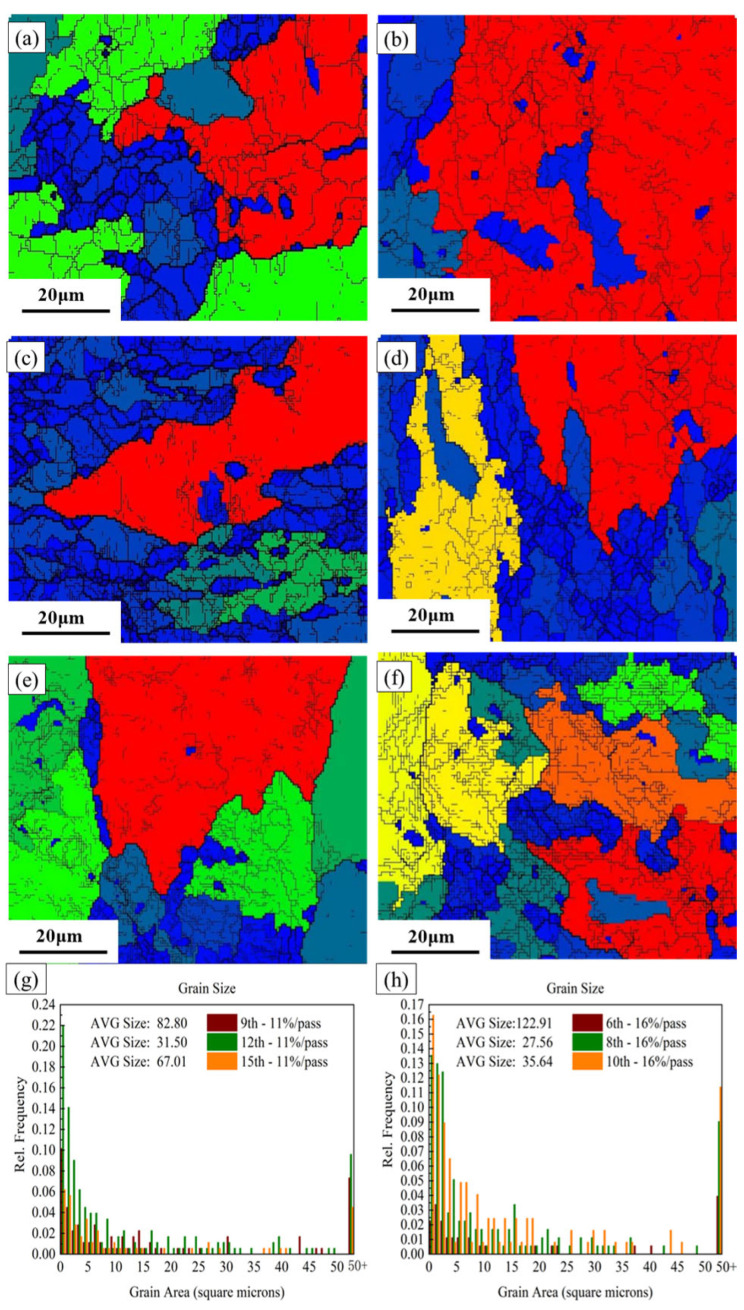
Grain structure (EBSD maps **a**–**f**) and grain size distributions (**g**,**h**) of the hot-rolled as-forged 7075 alloy obtained under different roll passes and reductions: (**a**) pass 9, 11%; (**b**) pass 12, 11%; (**c**) pass 15, 11%; (**d**) pass 6, 16%; (**e**) pass 8, 16%; (**f**) pass 10, 16%; (**g**) 11% reduction; (**h**) 16% reduction.

As shown in Figure 7g, the average grain size of the hot-rolled 7075 alloy with 11% pass reduction first decreases and then increases with the increasing rolling pass. The evolution of average grain size in the hot-rolled 7075 alloy demonstrates a distinct non-monotonic trend, which is critically dependent on both the number of rolling passes and the pass reduction, as quantified in Figure 7g,h. Under the 11% pass reduction schedule, the grain size decreases from 82.80 μm at the 9th pass to 31.50 μm at the 12th pass. For the 16% pass reduction, a similar refinement phase occurs but culminates earlier, reaching a minimum of 27.56 μm by the 8th pass (Figure 7e). The higher per-pass strain in the 16% schedule accumulates dislocation density and stored energy more rapidly per pass. Consequently, the critical strain for widespread DRX nucleation is reached sooner (by 8 passes) compared to the more gradual strain accumulation in the 11% schedule (by 12 passes). The finer initial recrystallized grains in the 16% condition (27.56 μm vs. 31.50 μm) further confirm the enhanced refinement efficiency of a higher pass reduction. With continued rolling beyond these points to the 15th and 10th pass for the 11% and 16% schedules, respectively, grain coarsening is observed, with sizes increasing to 67.01 μm and 34.22 μm. This reversal marks a shift in the dominant mechanism from strain-induced DRX to thermal-driven grain growth. The higher per-pass deformation in the 16% schedule generates more significant adiabatic heating, elevating the instantaneous temperature during deformation. Concurrently, the longer total time at high temperature associated with a greater number of passes in the 11% schedule provides an extended duration for thermal exposure.

Therefore, the observed non-monotonic grain size evolution is a direct outcome of the competition between strain accumulation and thermal exposure. The pass reduction dictates the intensity of each per-pass deformation and heat generation, while the number of passes controls the total accumulated strain and thermal history. The 16% reduction promotes faster, more intense refinement but also introduces greater thermal driving force for subsequent coarsening, precisely illustrating how these two key process parameters govern the microstructural trajectory.

**Figure 8:**
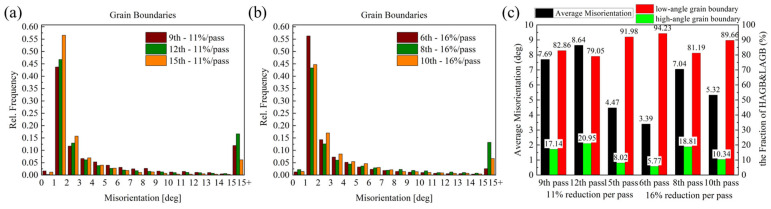
Statistical distribution of grain misorientation in the hot-rolled 7075 alloy under different reduction conditions: (**a**) 11%; (**b**) 16%; (**c**) average misorientation angle and low-angle/high-angle grain boundary content.

As illustrated in Figure 8a,b, the statistical distribution of grain misorientation angles in hot-rolling 7075 aluminum alloy under different pass reductions demonstrates that LAGB constitute the predominant type across all deformation conditions, with only a limited presence of HAGB exceeding 15°. Combined with the proportion of LAGBs and HAGBs shown in Figure 8c, it can be observed that as the deformation increased, the proportion of LAGBs exhibited a trend of first decreasing and then increasing. When the deformation reached its maximum, the proportion of LAGBs with misorientation angles below 15° was approximately 90% for both pass reduction conditions. This microstructural characteristic results from the material’s high stacking fault energy, which facilitates dislocation cross-slip and climb essential processes for Dynamic Recovery (DRV). During hot rolling, plastic deformation generates dense dislocation tangles that reorganize into sub-grain boundaries through polygonization, thereby increasing the LAGB density. Concurrently, the specific thermomechanical processing parameters including pass reduction and strain rate maintain a deformation storage energy below the critical threshold required for extensive dynamic recrystallization (DRX). Consequently, the microstructure becomes dominated by sub-grains separated by LAGBs, with HAGBs mainly originating from pre-existing grain boundaries and limited local recrystallization, reflecting the competing effects between work hardening and restoration mechanisms during processing. Meanwhile, Figure 8c reveals that the average grain orientation spread during hot rolling of the wrought 7075 alloy follows a non-monotonic evolution, characterized by an initial increase followed by a subsequent decrease as the number of rolling passes rises. This phenomenon is fundamentally governed by the competing effects between dislocation accumulation and the thermally activated processes of dynamic recovery and recrystallization. In the initial phase of hot rolling, the progressive accumulation of plastic deformation with increasing pass number results in a substantial increase in dislocation density. The resulting dislocation networks raise the density of low-angle boundaries, thereby elevating the average orientation spread. At this stage, work hardening dominates the microstructure evolution, while dynamic recovery remains inadequate to fully alleviate the dislocation proliferation. With further accumulation of rolling passes, the stored deformation energy reaches a threshold sufficient to activate widespread dynamic recrystallization. This process leads to the nucleation and growth of strain-free equiaxed grains, which progressively replace the heavily dislocated deformation substructure. Owing to their relatively uniform internal orientation, these recrystallized grains contribute to a notable reduction in the overall average orientation spread. The transition in the dominant mechanism from work hardening to dynamic recrystallization thus accounts for the non-monotonic variation in grain orientation spread.

**Figure 9:**
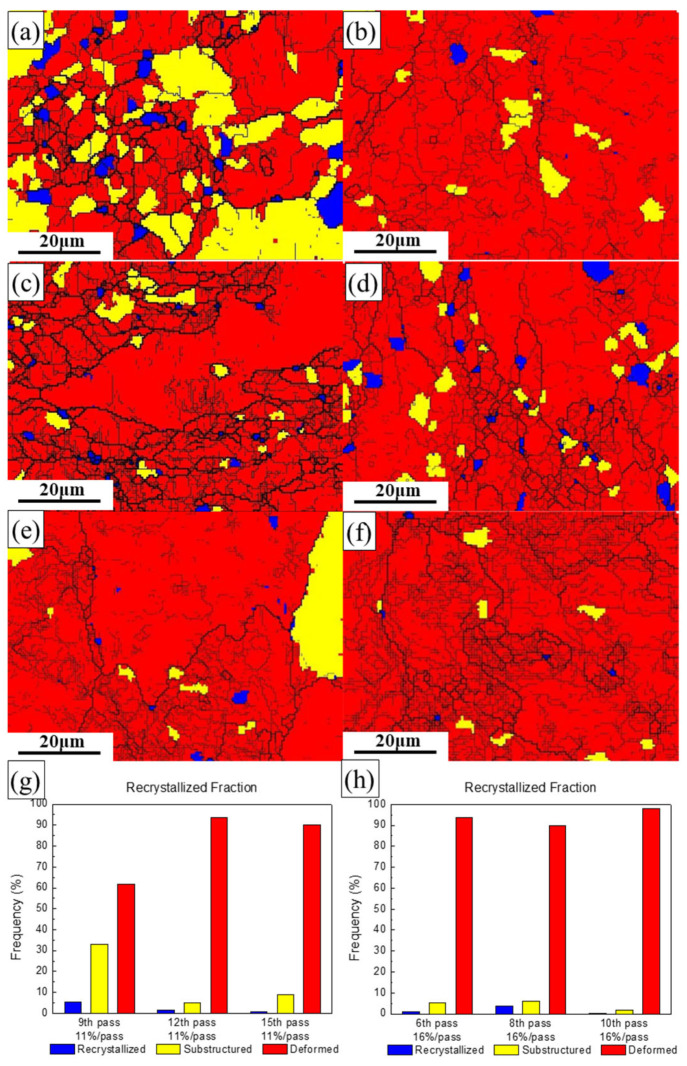
Evolved microstructures (recrystallized, substructured, and deformed) of the hot-rolled 7075 alloy as a function of roll pass and reduction: (**a**) 9th pass, 11%; (**b**) 12th pass, 11%; (**c**) 15th pass, 11%; (**d**) 6th pass, 16%; (**e**) 8th pass, 16%; (**f**) 10th pass, 16%; (**g**) summary for 11% reduction; (**h**) summary for 16% reduction.

Figure 9 shows the grain morphology of EBSD of hot-rolled 7075 alloy a with different rolling passes and pass reductions and presents the recrystallized, substructured, and deformed grains of the 7075 alloy after hot rolling with different rolling passes and pass reductions. The red, blue, and yellow colors represent deformed microstructures, recrystallized microstructures, and substructures, respectively. The results indicate that the microstructure of the 7075 alloy after the 9th pass of hot rolling with an 11% pass reduction consists of deformed, recrystallized, and substructured regions, which are interwoven as shown in Figure 9a. As the rolling pass increases to 12, the deformed grains increase significantly, while substructured and recrystallized grains decrease sharply and are sparsely distributed (Figure 9b). When the rolling pass further increases to 15, the microstructure becomes dominated by deformed grains, with substructures nearly disappearing and a small amount of recrystallized uniformly distributed within the deformed matrix (Figure 9c). In contrast, after only 6 passes of hot rolling with a 16% pass reduction, the 7075 alloy exhibits a predominantly deformed microstructure, with only limited recrystallized and substructured regions (Figure 9d–f). This demonstrates that under the same total reduction, increasing the pass reduction enhances the severity of plastic deformation, significantly promoting the formation of deformed microstructures while retarding dynamic recrystallization [37].

The observed microstructural evolution, where increased pass reduction promotes deformed microstructures while retarding dynamic recrystallization, is governed by the competing mechanisms of strain accumulation and thermal activation in high stacking-fault energy 7075 aluminum alloy [38]. The intensified plastic deformation per pass generates high dislocation density that rapidly reorganizes into substructures through dynamic recovery, which concurrently consumes the stored energy required for recrystallization nucleation. This dominant dynamic recovery mechanism, enhanced by the material’s inherent capacity for dislocation cross-slip and climb, effectively limits the driving force for complete recrystallization, resulting in a microstructure dominated by deformation features with limited recrystallized regions despite the increased strain severity.

**Figure 10:**
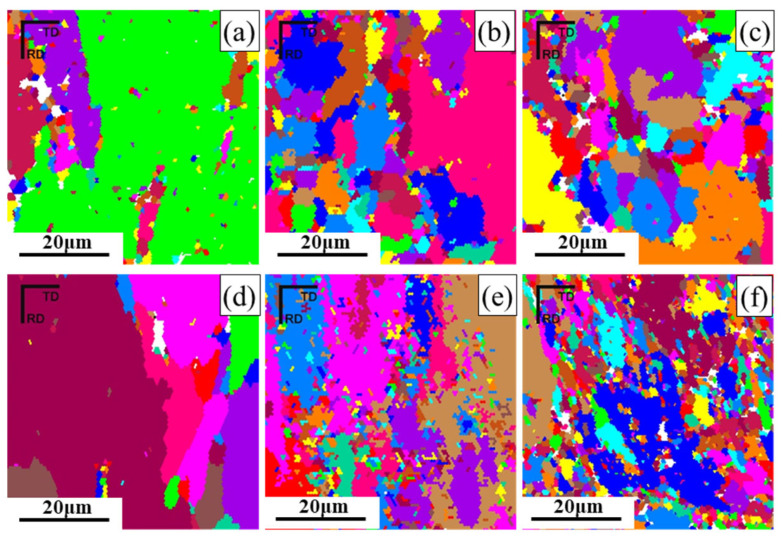
Grain misorientation distribution maps of the hot-rolled 7075 alloy as a function of roll pass and reduction: (**a**) 9th pass, 11%; (**b**) 12th pass, 11%; (**c**) 15th pass, 11%; (**d**) 6th pass, 16%; (**e**) 8th pass, 16%; (**f**) 10th pass, 16%.

Figure 10 presents the EBSD grain morphology of 7075 alloy after hot rolling with different roll passes and pass reductions. The results demonstrate that the severe plastic deformation during hot rolling results in a microstructure composed of elongated and equiaxed grains. The extensive plastic deformation induces work hardening in the hot-rolled 7075 alloy, enhancing its mechanical properties through dislocation generation. The elongated grains aligned along the rolling direction indicate consistent grain orientation. As observed in Figure 10a, after the 9th pass with 11% pass reduction, fine equiaxed grains begin to form at the grain boundaries of the microstructure. While most equiaxed grains are distributed along elongated grain boundaries, some are also present within the elongated grains. This phenomenon confirms the occurrence of dynamic recrystallization, which refines the grains and thereby improves the mechanical properties of the material, while simultaneously reducing work hardening effects through the decrease in dislocation density and sub-grains. In contrast, after the 6th pass with 16% pass reduction, no significant equiaxed grains resulting from dynamic recrystallization are observed in the microstructure. It is not until the deformation further increases to the 8th pass that a substantial number of equiaxed grains appear. This indicates that higher pass reduction delays the onset of dynamic recrystallization until greater cumulative deformation is achieved, consistent with the conclusions drawn from Figure 9. Furthermore, it can be noted that in the final stage of hot rolling (i.e., the 15th or 10th pass), the microstructure under 16% pass reduction with higher strain rate contains more equiaxed grains and exhibits finer grain size.

### 3.3. Texture Evolution of the Hot-Rolled Forged 7075 Alloy with Different Pass Reduction

**Figure 11:**
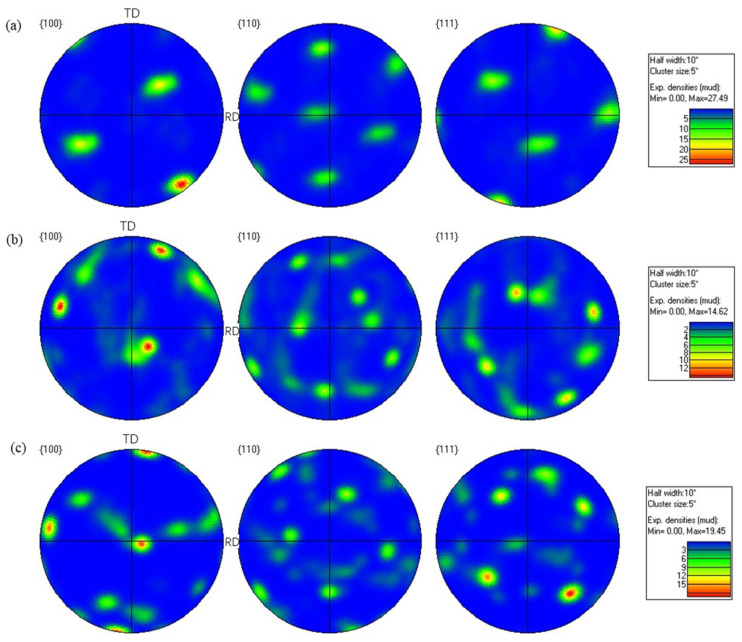
Texture images of the forged 7075 hot-rolled plate with 11% pass reduction: (**a**) 9th pass; (**b**) 12th pass; (**c**) 15th pass.

As is well known, the rolling process often induces texture formation in many metallic materials, which can substantially influence the mechanical properties of the metallic materials [39]. Figure 11 shows the pole figures of the hot-rolled 7075 alloy with 11% pass reduction. As shown in Figure 11, the texture intensity on the (100) crystal plane is stronger than those on the (110) and (111) crystal planes. The maximum texture intensity reaches 27.49 after the 9th rolling pass. However, when the rolling pass increases from 9 to 12, the maximum texture intensity decreases to 14.62. With further increase in the rolling pass from 12 to 15, the texture intensity increases again, reaching a maximum value of 19.45 mud at the 15th pass. The highest texture intensity among the three rolling passes corresponds to the 9th pass. In the initial stage of hot rolling (9th pass), plastic deformation is primarily accommodated by dislocation slip, leading to crystal lattice rotation into specific stable orientations to accommodate the imposed strain, thereby developing a strong deformation texture. In face-centered cubic (FCC) aluminum alloys such as the hot-rolled wrought 7075 alloy, typical rolling textures include Copper {112}<111>, {123}, and similar components [40]. These texture orientations develop pronounced peaks along the β-fiber [41], resulting in a maximum texture intensity of 27.49 observed on the (100) pole figure.

**Figure 12:**
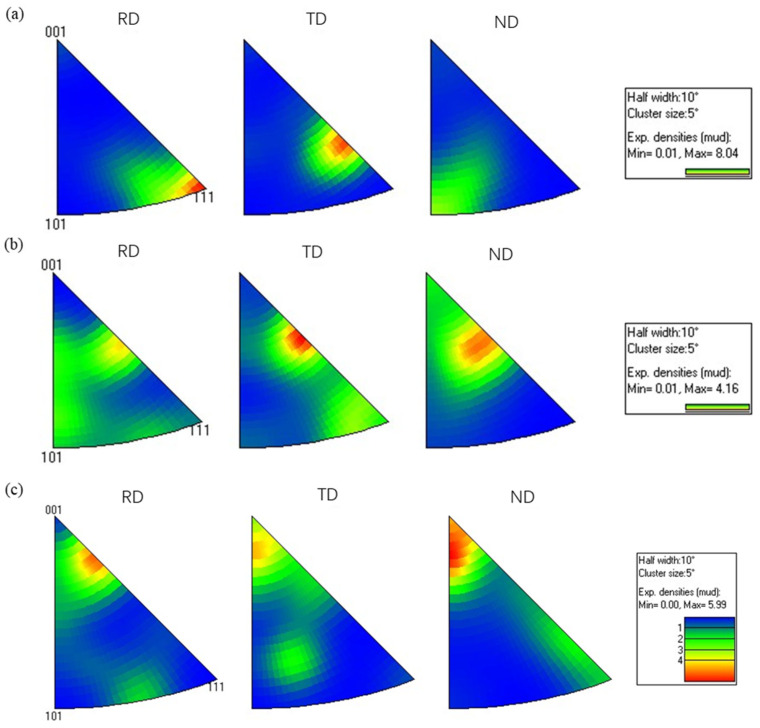
Inverse pole figure (IPF) of the hot-rolled 7075 alloy with 11% pass reduction (**a**) 9th pass; (**b**) 12th pass; (**c**) 15th pass.

The inverse pole figure (IPF) of the hot-rolled 7075 alloy is presented in Figure 12, revealing the influence of rolling passes on texture evolution. As shown in Figure 12, the texture generated by the hot rolling process is distributed in the rolling direction (RD), transverse direction (TD), and normal direction (ND). When the rolling pass was 9, the maximum texture intensity of 8.04 occurred in the <111> crystal orientation along the rolling direction (Figure 12a). As the rolling pass increased from 9 to 12, the texture intensity decreased significantly, and the texture distribution exhibited a notable shift from the rolling and transverse directions toward the transverse and normal directions. At the 15th rolling pass, the highest texture intensity of 5.99 is observed in the <001> crystal orientation along the normal direction, indicating that the increase in rolling passes significantly alters the preferred crystal orientations. The observed texture evolution, transitioning from a strong <111>//RD deformation texture to a weakened and reoriented state and finally to a dominant <001>//ND recrystallization texture with increasing rolling passes, is fundamentally governed by the shifting dominance between strain-induced texture formation and thermally activated restoration mechanisms. Initially, dislocation slip and crystal rotation under plastic deformation establish strong rolling texture components along the β-fiber. As strain accumulates, the activation of dynamic recovery and the nucleation of randomly oriented grains during dynamic recrystallization randomize the microstructure, weakening the initial texture.

**Figure 13:**
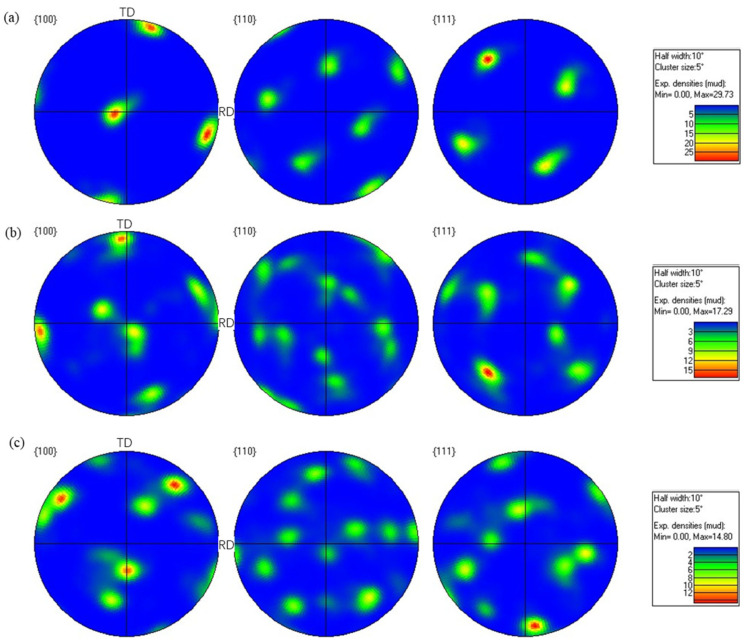
Pole figure of the forged 7075 hot-rolled plate with 16% pass reduction: (**a**) 6th pass; (**b**) 8th pass; (**c**) 10th pass.

**Figure 14:**
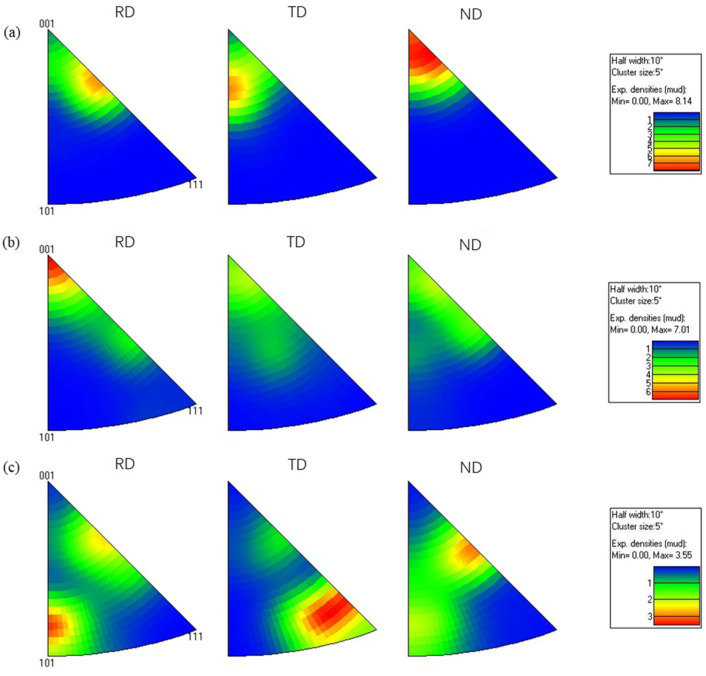
Inverse pole figure (IPF) of the forged hot-rolled 7075 alloy with 16% pass reduction: (**a**) 6th pass; (**b**) 8th pass; (**c**) 10th pass.

Figure 13 and Figure 14 present the pole figure and IPF of the hot-rolled wrought 7075 alloy with 16% pass reduction, respectively. As shown in Figure 13, the (100) and (111) crystal planes exhibited relatively high texture intensities. With increasing rolling passes, the maximum texture intensity gradually decreased from 29.73 to 14.80. This indicates that under severe plastic deformation, the proliferation of dislocations gradually weakens the texture organization with increasing strain. This phenomenon further promotes the formation of sub-grains and ultrafine-grained microstructure, which is consistent with the development of numerous fine equiaxed grains previously observed in Figure 10f. Additionally, the inverse pole figure in Figure 14 further demonstrates that when the rolling passes increased from 6 to 10, the maximum texture intensity decreased from 8.14 to 3.55. After the final pass with 16% pass reduction, the grain orientation exhibited a relatively dispersed distribution. A direct comparison between the texture evolution under 11% and 16% pass reductions clarifies the critical role of this parameter. In the initial stages (9th pass at 11% and 6th pass at 16%), both conditions develop strong textures (max intensities of 27.49 and 29.73, respectively). However, with progressive rolling, the texture intensity under the 16% reduction shows a pronounced and monotonic decreasing trend (from 29.73 to 14.80), culminating in a more randomized orientation distribution. In contrast, the texture evolution at 11% reduction is non-monotonic, first weakening significantly (27.49 to 14.62) and then strengthening again (to 19.45). This distinct behavior demonstrates that the higher pass reduction (16%) more effectively promotes grain orientation randomization and results in a weaker final texture. This is attributed to the more severe plastic deformation per pass, which generates higher dislocation density to accelerate dynamic recovery and recrystallization nucleation, while the associated adiabatic heating provides a stronger thermal driving force. Together, these mechanisms disrupt the initially formed deformation texture more rapidly and thoroughly.

During continuous hot rolling of aluminum alloy sheets, the sustained plastic deformation along the rolling direction typically induces numerous transverse textures, resulting in anisotropic material properties. This texture-induced anisotropy significantly influences the mechanical performance, though other factors including work hardening and dynamic recrystallization concurrently govern the microstructural evolution and mechanical properties during hot rolling. As shown in Figure 3, under constant total reduction, decreasing the number of rolling passes while increasing the pass reduction enhances the final mechanical properties of the rolled sheet. Moreover, the 16% pass reduction condition demonstrates reduced mechanical anisotropy compared to the 11% pass reduction. This can be attributed to the suppression of dynamic recrystallization by higher strain rates during hot working [42], leading to finer recrystallized grains (Figure 10) and consequently improved mechanical properties. Simultaneously, the adiabatic heating effect induced by severe plastic deformation promotes dynamic recrystallization [43], thereby weakening texture intensity and reducing material anisotropy. The texture comparison above provides direct microstructural evidence: the weaker and more dispersed texture resulting from the 16% reduction is a fundamental reason for its lower mechanical anisotropy.

### 3.4. Microstructure and Mechanical Properties of 7075 Hot-Rolled Plate After Heat Treatment

**Figure 15:**
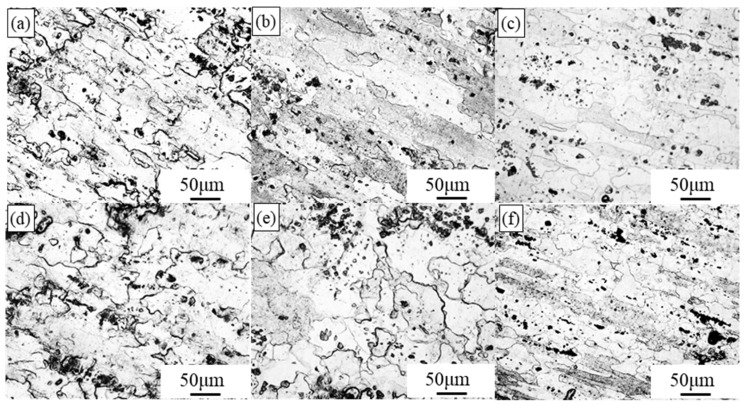
Optical microstructure of hot-rolled forged 7075 aluminum alloy plates with different pass reductions after heat treatment: (**a**) T6-11% pass reduction; (**b**) T7-11% pass reduction; (**c**) RRA-11% pass reduction; (**d**) T6-16% pass reduction; (**e**) T7-16% pass reduction; (**f**) RRA-16% pass reduction.

**Figure 16:**
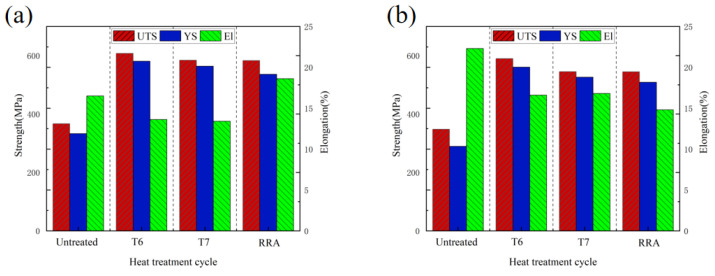
Mechanical properties of hot-rolled forged 7075 aluminum alloy sheets under different heat treatment schemes: (**a**)11% pass reduction; (**b**)16% pass reduction.

Figure 15 and Figure 16 present the optical microstructures and mechanical properties of hot-rolled wrought 7075 plates subjected to different heat treatments. Significant enhancements in YS and UTS are observed after three distinct heat treatment processes, though with a slight reduction in elongation compared to the initial wrought condition. Three distinct heat treatment schedules, namely T6, T7, and RRA (Retrogression and Re-Aging), were employed.

Three distinct schedules were employed:

1. The T6 treatment parameters consisted of solution treatment at 470 °C for 1 h followed by water quenching and subsequent artificial aging at 120 °C for 24 h.

2. The T7 treatment involved solution treatment at 470 °C for 1 h, water quenching, and a two-step aging process: first at 110 °C for 6 h and then at 140 °C for 16 h.

3. The RRA treatment comprised solution treatment at 470 °C for 1 h, water quenching, initial aging at 120 °C for 24 h, followed by a retrogression step at 190 °C for 15 min with water quenching, and a final re-aging at 120 °C for 24 h. Water quenching was applied immediately after the solution treatment stage for all three heat treatment schedules.

The T6 treatment demonstrated the most pronounced strengthening effect. Notably, plates hot-rolled with the 16% pass reduction consistently exhibited superior mechanical properties across all three heat treatment conditions compared to those rolled with 11% reduction. For instance, after T6 treatment: The 11% pass reduction plates achieved a YS of 560.7 MPa, UTS of 589.8 MPa, and elongation of 16.6%. The 16% pass reduction plates reached higher strengths of 580.9 MPa (YS) and 607.5 MPa (UTS), with an elongation of 13.62%. This performance optimization in the 16% condition is attributed to a synergistic effect of two strengthening mechanisms. Firstly, precipitation strengthening during aging played a dominant role. As revealed in Figure 15, T6-treated specimens contained a markedly higher density of fine, uniformly distributed precipitates compared to the T7 and RRA treatments. These precipitates effectively impede dislocation motion, thereby significantly increasing strength. Secondly, this was complemented by enhanced grain refinement strengthening from the prior hot rolling. The higher 16% pass reduction produced a finer initial grain structure (as shown in previous microstructural analysis). According to the Hall-Petch relationship, this finer grain size contributes additional strength by increasing the total area of grain boundaries, which act as barriers to dislocation propagation. The combined action of these two mechanisms explains the superior strength of the 16% pass reduction plates after T6 treatment.

## 4. Conclusions

(1) The ultimate tensile strength (UTS) and yield strength (YS) of hot-rolled 7075 aluminum alloy increase with the increase in total rolling reduction. The results show that when the total deformation exceeds 60%, the mechanical properties of hot-rolled sheets under both pass reductions (11% and 16%) are significantly enhanced, with the performance along the RD being markedly superior to that along the TD. For the 11% pass reduction schedule, the optimal mechanical properties were achieved at the 12th pass along the RD: UTS of 376.7 MPa, YS of 333.0 MPa, and EL of 17.6%.

(2) The rolling pass and pass reduction exert a decisive influence on microstructural evolution. The grain size does not vary monotonically during rolling, the average grain size first decreases due to refinement by dynamic recrystallization, and subsequently increases as a result of grain growth under thermal exposure. The prevalence of low-angle grain boundaries (LAGBs) reflects the dominant role of dynamic recovery in this high stacking fault energy material.

(3) Intense plastic deformation during hot rolling leads to texture formation. Under the 11% pass reduction condition, texture intensity exhibits a non-monotonic trend with increasing passes. In contrast, a higher pass reduction of 16% promotes more effective randomization of grain orientations and significantly weakens texture intensity. This texture weakening is induced by dynamic recrystallization disrupts pre-existing preferred orientations and contributes to reduced anisotropy in sheets rolled with 16% pass reduction.

(4) Heat treatment leads to a remarkable improvement in the strength of the hot-rolled sheets. The T6-treated plates exhibited a higher density of precipitated phases and a finer grain structure. Due to the synergistic effect of precipitation strengthening and grain refinement strengthening, these plates demonstrated the optimal mechanical properties. The sheets rolled with 16% pass reduction exhibit higher strength after T6 treatment compared to those with 11% reduction, achieving maximum UTS, YS and elongation values of 607.5 MPa, 580.9 MPa and 13.6%, respectively.

## Figures and Tables

**Table 1:** Chemical composition of 7075 commercial aluminum alloy (wt%).

Zn	Mg	Cu	Fe	Mn	Si	Cr	Ti	Al
6.0	2.3	1.56	0.47	0.27	0.26	0.17	0.03	Balance

**Table 2:** Rolling schedules with 11% pass reduction for the initial as-forged 7075 aluminum alloy (P1).

Rolling Pass	Pre-Rolling Thickness H (mm)	Post-Rolling Thickness h (mm)	Pass Reduction (%)	Insulation Time	Rolling Speed (m/min)	Cooling Method
1	21.00	20.00	4.76	1 h	5	Air-cooled
2	20.00	18.00	10.00	5 min	8	Air-cooled
3	18.00	16.10	10.56	5 min	8	Air-cooled
4	16.10	14.40	10.56	5 min	8	Air-cooled
5	14.40	12.90	10.42	5 min	8	Air-cooled
6	12.90	11.50	10.85	5 min	8	Air-cooled
7	11.50	10.28	10.61	5 min	8	Air-cooled
8	10.28	9.18	10.70	5 min	8	Air-cooled
9	9.18	8.18	10.89	5 min	8	Air-cooled
10	8.18	7.28	11.00	5 min	8	Air-cooled
11	7.28	6.48	10.99	5 min	8	Air-cooled
12	6.48	5.76	11.11	5 min	8	Air-cooled
13	5.76	5.11	11.28	5 min	8	Air-cooled
14	5.11	4.53	11.35	5 min	8	Air-cooled
15	4.53	4.00	11.70	5 min	8	Air-cooled

**Table 3:** Rolling schedules with 16% pass reduction for the initial as-forged 7075 aluminum alloy (P2).

Rolling Pass	Pre-Rolling Thickness H (mm)	Post-Rolling Thickness h (mm)	Pass Reduction (%)	Insulation Time	Rolling Speed (m/min)	Cooling Method
1	21.00	20.00	4.76	1 h	5	Air-cooled
2	20.00	16.90	15.50	5 min	8	Air-cooled
3	16.90	14.20	15.98	5 min	8	Air-cooled
4	14.20	11.95	15.85	5 min	8	Air-cooled
5	11.95	10.00	16.32	5 min	8	Air-cooled
6	10.00	8.35	16.50	5 min	8	Air-cooled
7	8.35	6.95	16.77	5 min	8	Air-cooled
8	6.95	5.80	16.55	5 min	8	Air-cooled
9	5.80	4.85	16.38	5 min	8	Air-cooled
10	4.85	4.00	17.53	5 min	8	Air-cooled

**Table 4:** Evolution of Mechanical Properties during Rolling.

Pass Reduction	Rolling Direction	Property	After 3 Passes	After Final Passes	Net Increase
11	RD	UTS (MPa)	256.7	347.9	91.2
		YS (MPa)	185.4	289.4	104
		EL (%)	22.1	22.2	0.1
	TD	UTS (MPa)	251.5	304.1	52.6
		YS (MPa)	219.9	289.6	69.7
		EL (%)	8.6	10.2	1.6
16	RD	UTS (MPa)	307.2	371.8	64.6
		YS (MPa)	238.5	337.9	99.4
		EL (%)	14.6	16.2	1.6
	TD	UTS (MPa)	278.3	337.4	59.1
		YS (MPa)	252.3	309.7	57.2
		EL (%)	6.3	10.5	4.2

## Data Availability

The original contributions presented in this study are included in the article. Further inquiries can be directed to the corresponding authors.

## References

[1] Li S., Yue X., Li Q., Peng H., Dong B., Liu T., Yang H., Fan J., Shu S., Qiu F. (2023). Development and applications of aluminum alloys for aerospace industry. J. Mater. Res. Technol..

[2] Yang J., Liu B., Shu D., Yang Q., Hu T. (2025). Vehicle giga-casting Al alloys technologies, applications, and beyond. J. Alloys Compd..

[3] Qin C., Jiang B., Hu M.-L., Wang Y., Xu H.-Y., Guo Y., Ji Z. (2024). Study on hot deformation behavior and dynamic recrystallization mechanism of recycled Al-Zn-Mg-Cu alloy. J. Mater. Res. Technol..

[4] Hua L., Hu X., Han X. (2020). Microstructure evolution of annealed 7075 aluminum alloy and its influence on room-temperature plasticity. Mater. Des..

[5] Zhu L., Li K., Yang X., He J., Fang J., Zhang Z., Guo M., Zhang J. (2024). Tailoring the formability and planar anisotropy of Al-Mg-Si-Cu-Zn alloys via cross hot rolling and two-stage cold rolling. J. Alloys Compd..

[6] Ma Y., Chen H., Zhang M.-X., Addad A., Kong Y., Lezaack M.B., Gan W., Chen Z., Ji G. (2022). Break through the strength-ductility trade-off dilemma in aluminum matrix composites via precipitation-assisted interface tailoring. Acta Mater..

[7] Kumar M. (2017). AW-7075-T6 sheet for shock heat treatment forming process. Trans. Nonferrous Met. Soc. China.

[8] Zhao T., Jiang Y. (2008). Fatigue of 7075-T651 aluminum alloy. Int. J. Fatigue.

[9] Mo T.Q., Chen Z.J., Li B.X., Wang P.J., Liu Q. (2019). Tailoring of interface structure and mechanical properties in ARBed 1100/7075 laminated composites by cold rolling. Mater. Sci. Eng. A.

[10] Zeng Q., Wen X., Zhai T. (2008). Texture evolution rate in continuous cast AA5052 aluminum alloy during single pass hot rolling. Mater. Sci. Eng. A.

[11] Zhang T., Wu Y., Gong H., Zheng X., Jiang S.-S. (2014). Effects of rolling parameters of snake hot rolling on strain distribution of aluminum alloy 7075. Trans. Nonferrous Met. Soc. China.

[12] Hidalgo P., Cepeda-Jiménez C.M., Ruano O.A., Carreño F. (2010). Influence of the processing temperature on the microstructure, texture, and hardness of the 7075 aluminum alloy fabricated by accumulative roll bonding. Met. Mater. Trans. A.

[13] Bagheripoor M., Bisadi H. (2011). Effects of rolling parameters on temperature distribution in the hot rolling of aluminum strips. Appl. Therm. Eng..

[14] Wang L., Yu H.S., Lee Y.S., Kim H.-W. (2015). Hot tensile deformation behavior of twin roll casted 7075 aluminum alloy. Met. Mater. Int..

[15] Sherstnev P., Melzer C., Sommitsch C. (2012). Prediction of precipitation kinetics during homogenisation and microstructure evolution during and after hot rolling of AA5083. Int. J. Mech. Sci..

[16] Takahashi R. (2001). State of the art in hot rolling process control. Control Eng. Pract..

[17] Meng L., Ding J., Li X., Cao G., Li Y., Zhang D. (2024). Novel shape control system of hot-rolled strip based on machine learning fused mechanism model. Expert Syst. Appl..

[18] Tao H., Liang X., Che Y., Zhou R., Wang L., Li H. (2024). Optimization of hot-rolling parameters for a powder metallurgy Ti-47Al-2Cr-2Nb-0.2W alloy based on processing map and microstructure evolution. J. Mater. Res. Technol..

[19] Ren J., Meng H., Yang M., Wang R., Peng C., Sun Y., Chen Y., Yang K., Song G. (2024). Strengthening Mg-8Li-3Al-2Zn-1Gd-0.2Zr alloy by combining hot extrusion and cold rolling. J. Mater. Res. Technol..

[20] Wang K., Li J., Stoughton T.B., Carsley J.E., Carlson B.E. (2018). Effect of preform annealing on plastic anisotropy of an age-hardenable Al-Mg-Si alloy. J. Mech. Work. Technol..

[21] Zhang K., He Q., Rao J.H., Wang Y., Zhang R., Yuan X., Feng W., Huang A. (2021). Correlation of textures and hemming performance of an AA6XXX aluminium alloy. J. Alloys Compd..

[22] Chen Y., Li J., Tang B., Kou H., Xue X., Cui Y. (2015). Texture evolution and dynamic recrystallization in a beta titanium alloy during hot-rolling process. J. Alloys Compd..

[23] Shah V., Sedighiani K., Van Dokkum J.S., Bos C., Roters F., Diehl M. (2022). Coupling crystal plasticity and cellular automaton models to study meta-dynamic recrystallization during hot rolling at high strain rates. Mater. Sci. Eng. A.

[24] Wu Y., Liao H., Lü C. (2019). Dynamic precipitation and recrystallization in Al-12.5 wt%Si-0.6 wt%Mg-0.1 wt% Ti alloy during hot-rolling and their impacts on mechanical properties. J. Alloys Compd..

[25] Ahn C.-W., Lee M.-S., Lee D., Cho S., Kim D.H., Sohn S.S., Park C.-S. (2025). Effect of hot-rolling temperature and reduction ratio on tensile properties of B4C-cBN reinforced aluminum matrix composite. J. Alloys Compd..

[26] Wang D., Li H., Song X., Ren Y., Fan Q., Zhu X., Chen L., Wang Y., Gao W., Cao Z. (2023). The dynamic recrystallization behavior of the Ti-5.5Mo-7.2Al-4.5Zr-2.6Sn-2.1Cr titanium alloy during hot rolling based on macro-meso multiscale crystal plasticity finite element approach. Mater. Today Commun..

[27] Senthil K., Iqbal M.A., Chandel P., Gupta N.K. (2017). Study of the constitutive behavior of 7075-T651 aluminum alloy. Int. J. Impact Eng..

[28] Abolhasani A., Zarei-Hanzaki A., Abedi H.R., Rokni M.R. (2012). The room temperature mechanical properties of hot rolled 7075 aluminum alloy. Mater. Des..

[29] Cao Y., He L., Zhang L., Zhou Y., Wang P., Cui J. (2016). Effects of magnetic field and hot rolling on microstructures and properties of cryoECAPed 1050 aluminum alloy during annealing. Trans. Nonferr. Met. Soc. China.

[30] Fang J., Mo J., Li J. (2017). Microstructure difference of 5052 aluminum alloys under conventional drawing and electromagnetic pulse assisted incremental drawing. Mater. Charact..

[31] Pourboghrat F., Venkatesan S., Carsley J.E. (2013). LDR and hydroforming limit for deep drawing of AA5754 aluminum sheet. J. Manuf. Process..

[32] (2008). Standard Test Methods for Tension Testing of Metallic Materials Metric.

[33] Chen Q., Lei W., Ran X. (2025). Effect of unidirectional rolling and cross rolling on the microstructure and mechanical anisotropy of Mg-6.3Gd-3Li-2Zn-0.5Al alloy. J. Alloys Compd..

[34] Markushev M.V., Nugmanov D.R., Sitdikov O., Vinogradov A. (2018). Structure, texture and strength of Mg-5.8Zn-0.65Zr alloy after hot-to-warm multi-step isothermal forging and isothermal rolling to large strains. Mater. Sci. Eng. A.

[35] Liu F.C., Ma Z.Y., Zhang F. (2012). High Strain Rate Superplasticity in a Micro-grained Al-Mg-Sc Alloy with Predominant High Angle Grain Boundaries. J. Mater. Sci. Technol..

[36] Kapoor R., Kumar N., Mishra R.S., Huskamp C.S., Sankaran K.K. (2010). Influence of fraction of high angle boundaries on the mechanical behavior of an ultrafine grained Al-Mg alloy. Mater. Sci. Eng. A.

[37] Zhang C., Li K., Zhao S., Chang M., Jiang H., Wang Y., Ren Y., Zhang D. (2025). Microstructure evolution and mechanical properties of high-strength 7075 aluminum alloy during multi-pass hot radial forging process. J. Mater. Res. Technol..

[38] Liu M., Shan Z., Li X., Zang Y. (2023). Hot tensile deformation behavior and microstructure evolution of 7075 aluminum alloy sheet. J. Mater. Res. Technol..

[39] Tian Q.M., Yang Y., Tan Y., Xiang S., Zhao F., Ji X.M., Huang G.W. (2025). Synergistically enhancing the strength and ductility of TA15 titanium alloy through hot rolling and short-time annealing. J. Alloys Compd..

[40] Jayaganthan R., Brokmeier H.G., Schwebke B., Panigrahi S.K. (2010). Microstructure and texture evolution in cryorolled Al 7075 alloy. J. Alloys Compd..

[41] Zhang Y., Fan Z., Li Y., Zhong J., Pang S., Nagaumi H. (2023). Intermediate temperature tensile behavior and processing map of a spray formed 7075 aluminum alloy. J. Mater. Res. Technol..

[42] Yang H., Qian Z., Sun P., Yang H., Zheng S., Li M. (2024). Flow behavior and activation energy evolution of 7075-T6 Al alloy during hot deformation. J. Met..

[43] Ohkoshi S.I., Nakagawa K., Yoshikiyo M., Namai A., Imoto K., Nagane Y., Jia F., Stefanczyk O., Tokoro H., Wang J. (2023). Giant adiabatic temperature change and its direct measurement of a barocaloric effect in a charge-transfer solid. Nat. Commun..

